# Integrating ChatGPT to support children and adolescents with diabetes mellitus in South African healthcare: Perspectives of healthcare professionals and academics

**DOI:** 10.4102/phcfm.v18i1.5433

**Published:** 2026-08-22

**Authors:** Kabelo G. Chuma, Philangani T. Sibiya

**Affiliations:** 1 Department of Information Science, College of Human Sciences, University of South Africa, Pretoria, South Africa

**Keywords:** diabetes mellitus, artificial intelligence, ChatGPT, children and adolescents, South Africa

## Abstract

**Background:**

The increasing prevalence of diabetes mellitus among children and adolescents in South Africa presents a major public health challenge, compounded by limited resources, inadequate infrastructure and poor disease management. Given these challenges, innovative solutions such as ChatGPT are essential to offer immediate, personalised education and social support to children and adolescents with diabetes.

**Aim:**

The study explored the integration of ChatGPT to support children and adolescents with diabetes in South African healthcare.

**Setting:**

This study was conducted with experts working in various organisations and institutions across Gauteng province of South Africa.

**Methods:**

A qualitative research design was used, with semi-structured interviews conducted with paediatric endocrinologists, diabetologists, diabetes educators and senior academics through purposive and snowball sampling. Data were analysed using thematic analysis and ATLAS.ti version 23.

**Results:**

The findings of the study revealed four key themes: perceptions of ChatGPT (both positive and negative); ethical concerns related to data security, reliability and technology overreliance; factors influencing acceptance, including usability, trust and accuracy; and obstacles to integration, such as technological, social and financial barriers.

**Conclusion:**

The study underscores the critical need for policy and regulatory frameworks, infrastructure development and government support in facilitating ChatGPT integration in South African healthcare to improve diabetes care.

**Contribution:**

This study contributes to the broader discourse on digital health innovations and their role in enhancing chronic disease management among vulnerable populations in South Africa.

## Introduction

Diabetes mellitus (DM) is an escalating global health problem that affects millions of children and adolescents in the 21st century. Diabetes is a chronic medical condition marked by elevated levels of blood glucose (or blood sugar), which leads over time to serious damage to the heart, blood vessels, eyes, kidneys and nerves.^1^ It is caused by an absolute insulin deficiency, such as type 1, or by a relative deficiency, such as type 2. Among the different types of diabetes, type 1 diabetes (T1D) in children has become one of the most significant global public health concerns, imposing a growing burden on families and countries. Xie et al.^2^ affirm that the onset of T1D is an escalating global health concern among children, adolescents and young adults, particularly in countries with a low-to-middle and middle Social Development Index. Indeed, the prevalence of T1D in children is particularly significant in low-income African countries, where the condition is often under-recognised, undetected and inadequately treated.^3^ It is estimated that approximately 1.2 million children and adolescents around the world suffer from type 1 and type 2 diabetes, with the majority living in developing countries.^4,5^ Despite this, a growing number of diabetes mellitus cases remain undiagnosed, particularly in resource-constrained healthcare settings with limited access to medical services, resources and early detection services.

Given the context, children and adolescents are regarded as individuals aged between 0 years and 19 years, in accordance with global and South African paediatric classification.^6^ There is, however, a wide range of cognitive maturity, health literacy and decision-making capability among this age group. It is unlikely that young children can engage with generative artificial intelligence (AI) tools independently, so they will need assistance or guidance from parents, caregivers or healthcare professionals. Despite their variable ability to evaluate health information, older adolescents may interact with these tools more independently. Nonetheless, ChatGPT can directly support adolescents and indirectly support younger children through parents and caregivers. Tran et al.^7^ emphasised that although ChatGPT has the potential to provide accessible health information, its application does not guarantee age-appropriate content without the aid of guided input or system design. This underscores the need for age-sensitive adaptations such as the use of simplified language and the implementation of safeguards. Furthermore, there is a need for legal and ethical considerations regarding minors’ access to digital platforms in South Africa, particularly in relation to consent and data privacy.

In the African context, the incidence and prevalence of T1D are rising, with 1 211 900 children aged 0–19 years diagnosed with the condition, and an additional 149 500 new cases identified annually.^8^ Prior research has highlighted geographic variations in the incidence of T1D among children across different regions. For example, the incidence of T1D among children is reported as 2.4 per 100 000 in Ethiopia^9^ and 1.5 per 100 000 in Tanzania,^10^ while higher rates have been observed in countries such as Algeria (4.4 per 100 000) and Morocco (20 per 100 000).^4^ Similarly, incidence rates vary in other regions, including 25 per 100 000 in Saudi Arabia and 3.75 per 100 000 in Egypt,^11^ as well as 2.7 per 100 000 in Rwanda and 11.2 per 100 000 in Eritrea.^8^ These variations reflect differences in healthcare systems, diagnostic capacity and socioeconomic conditions across countries.

In South Africa, the prevalence of diabetes is on the rise among children and adolescents, particularly T2D, which has been linked to lifestyle and socioeconomic factors.^12,13,14^ In contrast, T1D is reported at a lower incidence rate, with Dhada and Blackbeard^4^ estimating that the incidence of T1D among children in South Africa aged 0–14 years is 0.8 per 100 000. These incidence rates of type T1D appear lower than those reported in other African countries. However, this may be because of an underdiagnosis of T1D, limited surveillance systems, and disparities in healthcare access. As a result of this, caution should be exercised when making direct comparisons between countries, as differences in the methods for collecting and reporting data may affect the results. Despite these limitations, Lesage et al.^15^ stress that there have been growing concerns about the rising prevalence of T1D and T2D in South Africa since the onset of the coronavirus disease 2019 (COVID-19) outbreak. As a result of these trends, there is still a need to improve early detection, disease management and support systems for children and adolescents with diabetes. According to Thomas et al.,^16^ South African children and adolescents living with diabetes face several challenges that make it difficult for them to manage their disease effectively. These challenges include a lack of access to healthcare services, a lack of social and family support, difficulty monitoring insulin levels and adhering to treatments, and psychological difficulties like anxiety and frustration. As a result, this affects long-term glycaemic control and health outcomes.

Abrahams et al.^17^ argue that the South African public healthcare system is under severe strain and is unable to provide support and necessary care for young people living with diabetes. Public sector hospitals are operating under substantial pressure with outdated infrastructure, limited resources, legacy systems and a shortage of paediatric endocrinologists, all of which hinder the provision of quality care, personalised education and social support for young patients living with diabetes. Moore et al.^18^ ascertain that children and young adolescents with T1D from disadvantaged backgrounds or marginalised communities of South Africa struggle with managing their diabetes because of inadequate health services, inaccessible medication and poor digital literacy. Nonetheless, these contextual barriers result in higher risks of complications, hospital readmissions and poor health and treatment outcomes among young people living with T1D. In response to these challenges, AI in healthcare emerged as a solution that has the potential to reshape how patients with diabetes are diagnosed, treated and monitored. Tran et al.^7^ underscore that AI can support clinical consultations by improving patient education, simplifying complex medical information and improving clinician communication with patients and caregivers. Mondal and Naskar^19^ argue that generative AI tools such as ChatGPT present a promising solution to supplement overburdened and vulnerable healthcare services by providing accessible information, diabetes education and self-management support for children and adolescents. Daungsupawong and Wiwanitkit^20^ and Jairoun et al.^21^ attest that ChatGPT offers a promising path towards enhancing diabetes care through early detection, personalised treatment and medical triage support for children and adolescents with diabetes, aiding healthcare providers and patients in making well-informed decisions.

Despite the growing presence of AI in healthcare, there is limited understanding of how healthcare professionals perceive the applicability of tools such as ChatGPT in the management of chronic conditions such as diabetes among children and adolescents in South Africa. Although several studies in South Africa have explored AI in medical diagnosis^22^ and AI-based diabetes prediction,^23^ there is a noticeable gap in the literature on ChatGPT for diabetes management. Therefore, this qualitative study sought to explore the perceptions of healthcare professionals and academics on the use of ChatGPT in supporting diabetes management among children and adolescents. The study contributes to the body of knowledge by providing insight into how ChatGPT, as a representative AI tool, may support clinical interactions and patient care, rather than examining direct use by patients themselves. This article aims to answer the following research questions:

- *What are the perceptions of diabetes experts and senior academics relating to ChatGPT in supporting children and adolescents with DM in South Africa?*
- *What are the ethical concerns surrounding the use of ChatGPT for supporting children and adolescents with DM in South Africa?*
- *What are the factors influencing the acceptance and use of ChatGPT for supporting children and adolescents with DM in South Africa?*
- *What are the obstacles to integrating ChatGPT in healthcare to support children and adolescents with DM in South Africa?*

## Research method and design

### Study design

The study was grounded in the interpretivist paradigm, drawing from social constructivism to frame its context and guide the research approach. The study adopted an exploratory qualitative design, focusing on an in-depth exploration of contextual and subjective experiences, opinions and attitudes of the participants about the integration of ChatGPT in healthcare to support children and adolescents living with diabetes in South Africa. This design was well-suited to the context of this study as a result of the limited and/or incomplete understanding and knowledge surrounding the integration of ChatGPT to support children and teenagers with diabetes in the South African context. The researchers selected ChatGPT as part of this study because of its widespread usage, accessibility and advanced natural language processing capabilities, which make it a reliable large language model for generating health-related information. Although other platforms exist, such as Gemini, Claude, Qwen3 and Microsoft Copilot, ChatGPT has been adopted and evaluated in relation to health information. Rather than focusing on other healthcare applications, the study focused on Large Language Model (LLM) for assessing the ability of generative AI to provide conversational and user-driven health information, in contrast to the structured and predefined functionalities of traditional health applications.

### Study setting

This study was conducted with experts working in various organisations and institutions across Gauteng province of South Africa. The Gauteng province was selected as the study location because it has a diverse healthcare and academic landscape, including both public and private hospitals and universities with health experts and academics specialising in paediatric diabetes and health technology, making it an ideal setting to explore the feasibility and impact of AI-driven healthcare support. The study was conducted over a period of 3 months, from July 2023 to September 2023, during which participants provided insights through interviews on the considerations of integrating ChatGPT into diabetes management for young patients.

### Sample and recruitment

Purposive and snowball sample techniques were used to recruit paediatric endocrinologists, diabetologists, diabetes educators and senior academics with expertise in paediatric diabetes and experience in health technology. A purposive sampling technique was ideal for selecting participants with specialised knowledge and experience in paediatric diabetes care and health technology. Furthermore, snowball sampling allowed recruited participants to recommend other specialists and academics within their network or area of specialisation. Prospective participants were screened for eligibility based on their professional experience and relevant expertise. The criteria for selecting participants included a minimum of 10 years of professional experience and expertise in paediatric diabetes care. Moreover, participants were required to demonstrate experience adopting health technology (e.g. electronic health records, mobile health applications or AI-based systems) in clinical or academic settings, including prior experience using, implementing, evaluating or recommending digital health tools. Individuals aged 18 years or younger and those with less than 10 years of professional experience were excluded from the study. A total of 16 participants were interviewed. As part of the eligibility screening, researchers asked participants to share their knowledge, awareness and familiarity with ChatGPT and other AI language models. Most participants reported having a basic knowledge and understanding of ChatGPT, particularly through academic platforms, professional discussions and exposure to AI-aided tools used in healthcare. Some participants indicated that they had interacted with ChatGPT to explore its potential for patient education, while others had only a theoretical understanding. Participants did not necessarily possess advanced technical expertise in AI, and familiarity with ChatGPT was not used as an inclusion or exclusion criterion. However, all participants had substantial professional experience in paediatric diabetes care and exposure to digital health technologies within clinical and/or academic settings. This background enabled them to provide informed, grounded perspectives on the potential integration of ChatGPT into diabetes management.

### Data collection

The data in this study were collected using semi-structured interviews, which allowed for a more detailed exploration of the participants’ experiences while allowing for the flexibility to examine themes as they emerged. To ensure a basic understanding among participants, researchers provided a brief explanation of the capabilities and typical functions of ChatGPT. Participants were not provided with any formal training or lectures during the recruitment process; therefore, their responses were based on their broader clinical experience, professional judgement, varying levels of prior exposure and personal experience with ChatGPT. Before the commencement of interviews, participants were also asked to share their prior experience and familiarity with ChatGPT. Most participants demonstrated a good level of understanding and familiarity with the tool, while others had basic awareness of AI applications in healthcare. This enabled the researchers to establish baseline familiarity and provided insight into how their experiences and knowledge shaped their responses and perceptions during the interviews. During the interviews, all participants were exposed for the first time to the specific research questions related to the integration of ChatGPT for diabetes management for children and adolescents in a South African context. None of them had previously participated in the research related to this topic. Data saturation was achieved by the 13th participant during the interviews. Three interviews were conducted beyond the point of saturation. At the time of the study, the interviews were conducted through Microsoft (MS) Teams and in-person with participants and lasted from 30 min to 40 min until the necessary information was obtained. For quality control, all interviews were electronically recorded using a digital Dictaphone and the MS platform.

### Data analysis

Audio recordings were transcribed verbatim and analysed using Atlas.ti version 23 for Windows. All the interviews were conducted in the English language. Thematic analysis was employed to analyse the qualitative data, resulting in the generation of themes and categories.

### Rigour

This study employed reflective thematic analysis. Data were analysed using semantic coding analysis, focusing on the meaning of participants’ accounts to identify, organise and describe patterns across the dataset in line with the exploratory aims of this study. The researchers followed the six-step thematic analysis approach by Braun and Clarke, including (1) data familiarisation, (2) initial categorisation, (3) identification, review, and refinement of themes, (4) compilation of the final report and (5) presentation of the results. In this study, the themes (1) perceptions of ChatGPT, (2) ethical concerns, (3) factors influencing acceptance and (4) obstacles to integration were inductively informed by research objectives guiding this study. Subsequently, the sub-themes and categories within each theme emerged inductively through the analysis of the interview transcript. This approach ensured theoretical coherence and that participants’ experiences and perspectives were captured consistently and meaningfully. The trustworthiness of the study was ensured through sharing transcripts and preliminary findings with participants to review and validate their interview transcripts. Member checking was conducted to enhance the credibility and accuracy of the findings by allowing participants to review, scrutinise and validate the interpreted data. Subsequently, the findings were presented in a way that reflects the same experiences of participants, enabling the results of the study to become meaningful and recognisably accurate to the individuals involved.

### Ethical considerations

Ethical clearance to conduct this study was obtained from the University of South Africa College of Human Sciences Research Ethics Committee (No. CA4_29112022_CREC-CHS_2022). Participants were approached in person or via email and telephone and were invited to voluntarily participate in the study. As the research focused on individuals rather than institutional perspectives, obtaining formal permission from their respective institutions was deemed unnecessary. This approach ensured that participants could provide independent insights without institutional influence, maintaining the integrity and authenticity of their responses. All participants provided written informed consent prior to data collection. During the course of the study, participants were assured that their personal identities would remain anonymous and that they could withdraw at any time without facing any penalties.

## Results

The study comprised 16 participants representing a diverse group of health professionals and academics with knowledge and experience in paediatric diabetes and health technology. The participants comprised nine females and seven males, holding postgraduate diplomas, master’s or doctoral degrees, with professional experience ranging from 10 years to 25 years. They were affiliated with various academic institutions and hospitals across South Africa. Table 1 depicts demographic characteristics of the participants.

**TABLE 1:** Demographic characteristics of the participants.

Codes	Role	Gender	Experience (years)	Field or discipline	Qualification	Specialisation
A1	Diabetes Health Specialist	Male	12	Clinical diabetes care	Master’s Degree	Artificial Intelligence and Machine Learning in Health
A2	Diabetologist	Female	25	Clinical medicine	Master’s degree	Technology and Diabetes
A3	Senior Lecturer	Male	18	Health Sciences	Doctoral degree	Artificial Intelligence in Healthcare
A4	Professor	Male	20	Health technology	Doctoral degree	Health Technologies and Diabetes
A5	Senior Lecturer	Female	10	Public Health	Doctoral degree	AI and Telemedicine in Healthcare
A6	Diabetes Educator	Female	15	Paediatric diabetes	Postgraduate diploma	Growth and Endocrine Disorders and Diabetes in Children and Adolescents
A7	Professor	Male	18	Biomedical Informatics	Doctoral degree	AI and Cognitive Computing in Healthcare
A8	Paediatric Endocrinologist	Female	14	Paediatric medicine	Master’s degree	Diabetes and Metabolic Endocrinology
A9	Diabetologist	Female	17	Clinical paediatrics	Postgraduate diploma	Diabetes Management for Children and Adolescents
A10	Associate Professor	Female	15	Health Sciences	Doctoral degree	Type 1 and 2 Diabetes and Technology
A11	Professor	Male	19	Health Informatics	Doctoral degree	AI- and Machine Learning in Healthcare
A12	Diabetes Educator	Female	23	Public Health	Master’s degree	Diabetes Management and Education
A13	Diabetologist	Male	11	Clinical paediatric	Doctoral degree	Diabetes Management for Children
A14	Senior Lecturer	Female	14	Health Technology	Doctoral degree	Technology in Healthcare
A15	Diabetologist	Female	20	Clinical medicine	Master’s degree	Diagnosis, Treatment, and Management of diabetes
A16	Endocrinologist	Male	10	Clinical endocrinology	Postgraduate Diploma	Diabetic Health Nutrition and Technology

AI, artificial intelligence.

The findings of the thematic analysis resulted in four major themes and 13 categories as illustrated in Table 2.

**TABLE 2:** Themes and categories emerged from interviews about the integration of ChatGPT to support children and adolescents with diabetes mellitus.

Themes	Categories	Sub-categories
1 Perceptions of ChatGPT for supporting children and adolescents with diabetes mellitus	1.1 Positive perceptions of ChatGPT (Perceived usefulness)	Convenience and accessibility Educational support for families Potential to reduce workload for healthcare providers
	1.2 Negative perceptions of ChatGPT (Perceived limitations)	Lack of emotional support and empathy Risk of misleading or inaccurate advice Inability to meet individualised demands
2 Ethical concerns surrounding the integration of ChatGPT to support children and adolescents with diabetes mellitus	2.1 Concerns about language barriers	Limited support for local languages English-dominance barriers
	2.2 Concerns about accuracy and reliability of information	Inaccurate or unvalidated information Misleading guidance
	2.3 Concerns about security, privacy and transparency	Risk of data breaches Fear of unauthorised sharing Lack of transparency
	2.4 Concerns about technology over-reliance	Overdependence on AI instead of clinical judgement
3 Factors influencing the acceptance and use of ChatGPT to support children and adolescents with diabetes mellitus	3.1 Ease of use and user acceptance	User friendliness Familiarity with ChatGPT functionalities
	3.2 Perceived trustworthiness	Accuracy and reliability of responses Transparency in how information is generated
4 Obstacles to integrating ChatGPT in healthcare to support children and adolescents with diabetes mellitus	4.1 Infrastructural limitations	Outdated hardware and software Reliance on the legacy system Poor internet connectivity
	4.2 Human and organisational barriers	Resistance to change Lack of AI related training and skills
	4.3 Financial constraints	Cost of implementation Limited financial and political support

AI, artificial intelligence.

The results of this study are arranged according to four themes.

### Theme 1: Perceptions of ChatGPT for supporting children and adolescents with diabetes mellitus

In interviews, most participants viewed ChatGPT integration in South African healthcare for parents of children and adolescents with diabetes positively, while a few had negative perceptions. This theme was categorised into positive and negative perceptions.

#### Category 1.1: Positive perceptions of ChatGPT (Perceived usefulness)

‘From point of view [*sic*], I see a lot of promise in using AI and ChatGPT in South African healthcare to support children with diabetes. ChatGPT could offer immediate, personalised responses to questions about diabetes management and help with educational content, which could be very reassuring for both children and their families.’ (A8, Paediatric Endocrinologist, Female)

‘I strongly believe that this AI tool can make it much easier for parents to assist their children and adolescents to manage their sugar diabetes levels, while will [*sic*] reducing the risk of the health complications and this tool can also assist healthcare providers to provide information and support to patients with diabetes and reduce the strain or burden on the South African healthcare.’ (A4, Professor, Male)

‘I think integrating ChatGPT in South African healthcare system could be a great educational resource for children and adolescents with type 1 or 2 diabetes … I mean it could help explain complex concepts in a way that’s easy for children, young adults, and parents at home to understand and make learning about diabetes more engaging and how they should manage their blood sugar and insulin levels by eating a balanced diet filled with whole grains, vegetables, legumes.’ (A14, Senior Lecturer, Female)

‘The government of South Africa must find a way to integrate AI tools such as ChatGPT to support not only children and adolescents but everyone in South Africa who has diabetes whether type 1 or type 2.’ (A15, Diabetologist, Female)

‘I strongly believe that integrating the ChatGPT in our healthcare system could serve as a valuable educational resource for families that have children and adults with diabetes and other chronic diseases. I have tested this tool several times and I am confident that it has the capability to provide clear, understandable information about diabetes management and this can possibly help parents and children better understand how to handle the condition. Moreover, I have realised that other suggestions from the ChatGPT require guidance from healthcare professionals or clinical staff for verification.’ (A5, Senior Lecturer, Female)

These quotations demonstrate that participants shared their perceptions that ChatGPT could possibly enhance diabetes education and provide accessible support to families. The responses illustrate the potential of ChatGPT in supplementing existing diabetes management practices and thereby aligning directly with the theme of positive perceptions.

#### Category 1.2: Negative perception of ChatGPT (Perceived limitations)

On the contrary, few participants during interviews expressed their negative perceptions of the integration of ChatGPT in healthcare for supporting children and adolescents with diabetes:

‘From my opinion, I don’t think it is a great idea to integrate the ChatGPT in South African healthcare system. It is important to remember that diabetes management often requires emotional support and empathy, of which ChatGPT cannot provide. The human intervention is essential in healthcare, and AI in this case might fail to meet the emotional and psychological needs of children and adolescents living with diabetes. In addition to this, I think this tool is not reliable because it can sometimes provide a misleading or incorrect information, and this could result in confusion or even harmful advice for patients with diabetes and other chronic conditions.’ (A12, Diabetes Educator, Female)

‘I don’t support the integration of ChatGPT particularly because this tool is lacking the ability to offer the individualised attention required by children and adolescents with diabetes. Each person’s situation varies, and a standard AI reply or suggestion may not adequately meet individual demands.’ (A1, Diabetes Health Specialist, Male)

The responses should be understood in the context of the interpretivist paradigm that underpins the study, in which participants drew upon their personal and professional experiences, their observations of systemic healthcare issues, and their exposure to emerging digital health innovations. The perspectives of these frontline clinicians and educators relate more to their practical experience and perception of potential rather than empirically established outcomes. Thus, their assertions were based on professional judgement, but not definitive information regarding the effectiveness of ChatGPT in diabetes management.

Moreover, there was a distinct difference between participants with information technology (IT)-related expertise and those with clinical expertise. Participants with prior experience and knowledge of generative AI tools such as ChatGPT were more confident in its ability to provide personalised and social support and education for diabetes management. In contrast, clinicians with limited or no exposure to ChatGPT expressed several ethical concerns, including the accuracy of information, patient safety and the risk of overreliance. Nonetheless, both participants were concerned about the ethical implications of ChatGPT and acknowledged that it can complement clinical expertise in managing diabetes and not replace it.

Overall, responses in this theme demonstrate concerns about ChatGPT, including emotional limitations, misinformation and inability to substitute personalised clinical judgement. As a result, these responses directly complement the theme by emphasising why some participants remained cautious about integrating generative AI tools in diabetes management.

### Theme 2: Ethical concerns surrounding the integration of ChatGPT to support children and adolescents with diabetes mellitus

During interviews, many participants voiced concerns about integrating ChatGPT to support children with diabetes, leading to the emergence of four key categories:

#### Category 2.1: Concerns about language barriers

Participants expressed concerns about language barriers when using ChatGPT in healthcare:

‘I fully support the idea of integrating the ChatGPT in healthcare; however, my concern is the issue of language. The language used by ChatGPT is English and this may create a barrier for most of the children and parents. For instance, people from rural communities or disadvantages background who doesn’t know or understand English language this might be a challenge for them to use and understand the information and suggestion from the ChatGPT.’ (A9, Diabetologist, Female)

‘I am concerned that ChatGPT may not be able to comprehend South African languages like Zulu, Sepedi, or Xhosa. It is crucial to keep in mind that ChatGPT may struggle to comprehend questions in languages other than English because not all users are fluent in English, which can be a limitation of natural language processing. If not, this might result in giving wrong guidance, endangering children, and teenagers with diabetes.’ (A2, Diabetologist, Female)

‘My main concern with the ChatGPT is that most of the children and adolescents might not understand English language and this can create confusions. I wish this tool was flexible enough or customised to accommodate other South African languages so that people can be able to understand information, suggestions or medical advice provided by ChatGPT.’ (A3, Senior Lecturer, Male)

#### Category 2.2: Concerns about accuracy and reliability of information

During the interviews, participants expressed concerns about accuracy and reliability when using ChatGPT in healthcare:

‘Although ChatGPT can offer useful information, I am worried about the accuracy and reliability of information. I am wondering if this tool will always provide accurate and reliable information that will help children and adolescents to manage diabetes. I think it is important l that this tool is regularly updated, maintained, and validated to ensure that it provides reliable advice for managing diabetes.’ (A5, Senior Lecturer, Female)

‘I am concerned about the precision of the medical and health guidance offered by ChatGPT. Regularly reviewing and updating the ChatGPT is particularly essential to guarantee accurate information aligned with current medical standards and needs of patients with diabetes and other chronic conditions. People shouldn’t rely on it too much too because information and advice from this tool must be verified and validated.’ (A1, Diabetes Health Specialist, Male)

‘Incorporating ChatGPT into healthcare is a great idea to enhancing patient interactions and elevating the standards of personalised care. Despite its potential to provide information, my greatest concern over this technology is the accuracy of information it provides. This is because certain information from this tool is not verified and there is possible that it can provide relatively inaccurate information or misleading advice, putting patients living with diabetes at risk.’ (A7, Professor, Male)

#### Category 2.3: Concerns about security, privacy and transparency

‘While AI technologies have great potential to enhance healthcare and empower patients, there are still significant security and privacy concerns that must be addressed. I’m particularly worried about the safety of personal information because parents and children living with diabetes might hesitate to share their health details with ChatGPT, fearing that their personal information could be misused or accessed by cybercriminals and hacktivists.’ (A10, Associate Professor, Female)

‘I am concerned that ChatGPT might not have strong and reliable security measures to safeguard or protect sensitive information about patients with diabetes. For instance, if this platform were to be hacked, this means that any personal and medical information, as well as patient privacy, could be compromised and exposed to unauthorised parties.’ (A6, Diabetes Educator, Female)

‘My concern with this platform is its lack of transparency. I’m uneasy about how the sensitive information of patients living with diabetes will be managed and protected, who will have access to it, and how it might be used in the future. In the digital age, it’s hard to fully trust any platform with personal information because it could potentially be compromised by anyone.’ (A15, Diabetologist, Female)

‘I’m concerned that information about children and adolescents with diabetes might be shared with third parties without their knowledge or consent, particularly if the terms and conditions are unclear or difficult to understand. While people can use these platforms to meet their health and medical needs, it’s crucial that they avoid sharing their personal and sensitive information, as this could put their privacy at risk.’ (A13, Diabetologist, Male)

#### Category 2.4: Concerns about technology over-reliance

‘These AI platforms can indeed transform the South African healthcare industry, but my biggest concern about these platforms, is that our healthcare professionals including doctors, physicians and nurses may over depend on the ChatGPT, leading to a potential decline in the quality of patient care. Our doctors and nurses may heavily rely on ChatGPT for making decisions about patients with diabetes and other chronic diseases instead of relying on their own expertise and knowledge, and this could result in less personalised and poor-quality healthcare outcomes.’ (A4, Professor, Male)

These quotations demonstrate ethical concerns pertaining to language barriers, data privacy, information accuracy and transparency. These concerns are consistent with this theme by emphasising the possibility that perceived risks could prevent the adoption of ChatGPT in clinical settings.

### Theme 3: Factors influencing the acceptance and use of ChatGPT to support children and adolescents with diabetes mellitus

Participants were asked to identify factors affecting its use in supporting children and adolescents with DM, leading to the emergence of four categories within this theme.

#### Category 3.1: Ease of use and user acceptance

**Usability and functionality**

‘I believe that individuals with chronic diseases would be more likely to accept ChatGPT if it is easy for them to use and user-friendly. For instance, if a system or tool is straightforward and simple to navigate, people are more inclined to develop an interest in using it.’ (A3, Senior Lecturer, Male)

‘Personally, I think that if ChatGPT proves to be highly effective in performing its intended tasks … such as providing relevant information or answering questions from people with diabetes and offering the necessary support they need … it could significantly influence their acceptance of the platform and build strong foundation of trust in AI-driven interactions.’ (A12, Diabetes Educator, Female)

#### Category 3.2: Perceived trustworthiness

**Trust and credibility**

‘Transparency and credibility are crucial for ChatGPT. I believe that if parents of children and adolescents with diabetes can understand how the platform works and see its effectiveness in providing support and validated information, this will enhance trust and credibility, making them more likely to accept it.’ (A10, Associate Professor, Female)

‘I believe that people will be more likely to accept and trust ChatGPT if it consistently generates reliable results that could effectively support children and adolescents in managing their blood sugar levels and dietary needs. Additionally, if this AI platform operates with transparency, it can significantly enhance trust among patients with diabetes in South Africa.’ (A5, Senior Lecturer, Female)

**Accuracy and reliability**

‘My overall impression is that people will only accept ChatGPT if it can deliver contextually accurate and insightful information, as well as reliable answers to a variety of health-related questions posed by patients with diabetes. For this platform to build trust and confidence among users, it’s essential that the information it provides is verified and cross-referenced with reliable sources.’ (A9, Diabetologist, Female)

‘I strongly believe that the acceptance of ChatGPT will largely depend on its accuracy in responding to users’ questions about diabetes and other health conditions. To foster trust and widespread adoption, the platform must not only deliver precise and reliable information but also provide detailed resources that cater specifically to the needs and preferences of children and adolescents with diabetes.’ (A11, Professor, Male)

**User familiarity and understanding**

‘My assumption is that if patients with diabetes become familiar with ChatGPT and understand how it works, they will be more likely to accept and use it whenever they need advice or information on monitoring their blood sugar levels, exercising, maintaining a healthy diet, and taking medications.’ (A2, Diabetologist, Female)

Based on these insights, it is evident that the acceptance of ChatGPT is influenced by usability, trust, accuracy and prior experience with generative AI tools. As a result, participants’ responses support this theme by demonstrating the conditions under which ChatGPT may be deemed credible and beneficial.

### Theme 4: Obstacles to integrating ChatGPT in healthcare to support children and adolescents with diabetes mellitus

During the interview, participants were asked to identify potential obstacles that could impede the integration of ChatGPT into the South African healthcare system. Three categories emerged from this theme.

#### Category 4.1: Infrastructural limitations

‘It is important to remember that integrating ChatGPT presents several challenges. For South Africa, in particular, I think incorporating this platform into healthcare systems may be difficult due to the poor and outdated infrastructure we have in our healthcare facilities. Specifically, outdated infrastructure could pose a significant obstacle for public hospitals attempting to use ChatGPT to support children and adolescents with diabetes.’ (A16, Endocrinologist, Male)

‘I think the integration of ChatGPT into healthcare will be particularly challenging due to the prevalence and overreliance on legacy systems and outdated software and hardware. In South Africa, many hospitals and clinics are still using legacy and fragmented systems that lack the advanced features and functionalities needed to support AI and other modern technologies. It is crucial for the government to prioritise the modernisation of health systems and applications to accommodate new technologies.’ (A8, Paediatric Endocrinologist, Female)

‘I believe that limited bandwidth and internet access are major obstacles. In South Africa, many healthcare facilities, particularly in rural and semi-rural areas of provinces like KwaZulu-Natal and Eastern Cape, lack stable network and reliable internet connections. In summary, the absence of dependable internet and advanced technological tools could pose a significant barrier to integrating ChatGPT into the healthcare system.’ (A14, Senior Lecturer, Female)

#### Category 4.2: Social obstacles

‘I firmly believe that resistance from healthcare professionals unfamiliar with AI technologies could be a major barrier to integrating ChatGPT. If doctors and nurses are not adequately trained, lack trust in the technology, or have insufficient knowledge about the platform, it could significantly impede the adoption of ChatGPT within our healthcare system.’ (A2, Diabetologist, Female)

‘In my opinion, I personally think that healthcare professionals, including doctors, nurses, and other practitioners, may resist using ChatGPT if they do not receive adequate training and skills. Without a clear understanding of how the system functions, there is likely to be significant scepticism and resistance.’ (A13, Diabetologist, Male)

#### Category 4.3: Financial constraints

‘Deploying and upkeeping AI technology such as ChatGPT may be overly costly, particularly in South African public hospitals facing constraints with resources.’ (A9, Diabetologist, Female)

‘From my perspective, cost is a major hurdle. I mean integrating and maintaining AI technologies like ChatGPT into health systems could be quite expensive, and in a resource-limited setting like South Africa, this could be particularly challenging. Nevertheless, this is especially true if there’s no financial and political support from the government or international partners.’ (A7, Professor, Male)

‘I think the integration of ChatGPT into the South African healthcare system could be hindered by many challenges, for instance insufficient financial resources, poor infrastructure … as well as inadequate technical skills.’ (A3, Senior Lecturer, Male)

Based on these perspectives, it is evident that infrastructural limitations, resource constraints, lack of digital skills and resistance to change hinder the adoption of ChatGPT in the healthcare setting. It is clear from the quotations that they support the theme by illustrating how these obstacles could hinder practical implementation.

## Discussion

The findings revealed the potential benefits of ChatGPT as a means for enhancing communication during consultations and as a source of supplementary information based on the perspectives of clinicians and academics. These findings align with Dey^24^ who emphasised the ability of ChatGPT to offer continuous health education. Participants believed that ChatGPT could help families better understand diabetes, assist with blood sugar control and reduce the burden on healthcare providers. Moreover, there was consensus that ChatGPT could advance diabetes education by making it more engaging and accessible, provided that government support facilitates its integration.

However, participants expressed concern about the reliability and implications of AI-generated information. This concern is supported by Al-Anezi^25^ who cautioned against overreliance on AI because of its inability to offer human-like emotional understanding. Accuracy and reliability were also major concerns. Some participants feared that incorrect or misleading information could lead to harmful health decisions, particularly for chronic conditions like diabetes. These concerns align with Chatelan et al.^26^ who stressed that ChatGPT may generate inaccurate and unreliable treatment recommendations. However, other scholars, such as Huang et al.^27^ and Sng et al.^28^, argue that ChatGPT can provide reliable health information when properly validated. In this digital era, many AI-driven healthcare solutions have addressed these concerns through incorporating a variety of expertise, including medical consultants, patient educators and health counsellors to monitor and validate that the health information provided to patients is accurate. It is evident from dedicated medical applications that their content has been clinically reviewed and aligned with established guidelines. Nevertheless, these structured validation processes may not be integrated into large language models such as ChatGPT, which are based on probabilistic text generation rather than real-time clinical oversight. Therefore, using large language models in healthcare requires reliable safeguards, validation mechanisms and professional oversight.

Linguistic accessibility emerged as another challenge. Many participants expressed concerns about the reliance of ChatGPT on the English language, which could exclude non-English-speaking users, particularly in rural South African communities. This linguistic barrier could hinder equitable access to healthcare support. Security and data privacy were also critical concerns. Although data privacy concerns are often associated with AI technologies, the risks in this context are related to how clinicians interact with tools such as ChatGPT. For instance, the entry of identifiable patient information into AI platforms could raise concerns regarding confidentiality. However, such risks can be minimised when used appropriately as a tool for general information without sharing sensitive patient information. Participants further stressed the importance of adhering to ethical guidelines and professional standards when integrating AI into clinical practice. Beyond the South African context, international literature has demonstrated the ethical concerns surrounding the use of ChatGPT to support diabetes care and other chronic diseases. A series of studies conducted in Asia, North America and Europe indicated several ethical and legal concerns associated with generative AI tools in DM, including data privacy and security risks, deficiencies in transparency and interpretability, educational and language disparities, inaccurate responses, responsibility and copyright concerns and the risk of misinformation.^29,30,31^

The ethical concerns mentioned by participants in this study are consistent with those raised by other scholars, suggesting that ethical issues related to trust, transparency and responsible use of ChatGPT are not unique to South Africa, but are shared across diverse healthcare systems. Nevertheless, in resource-constrained settings such as South Africa, these ethical risks can be exacerbated by infrastructural limitations, linguistic diversity and disparities in digital literacy, underscoring the need for regulatory approaches that are sensitive and internationally informed. Participants expressed scepticism about the ability of ChatGPT to safeguard patient data, fearing potential cybersecurity breaches and a lack of transparency in data management. These concerns align with Alawida et al.,^32^ who warned that AI-driven health platforms could expose sensitive medical data to cyber threats and fraud.

Several key factors were identified as influencing the acceptance of ChatGPT in diabetes management. Participants emphasised that user-friendliness and ease of navigation are crucial, particularly for individuals managing chronic conditions. This aligns with Zhang and Zhao,^33^ who found that perceived usefulness and ease of use significantly influence AI adoption in healthcare. Trust in ChatGPT was seen as contingent on its ability to provide accurate, verified and cross-referenced information. These findings are consistent with Ittarat et al.^34^ and Triplett,^35^ who found that familiarity with AI tools enhances trust and acceptance. Furthermore, participants indicated that the more users, particularly parents of children with diabetes, understand how to use ChatGPT for tasks like blood sugar monitoring and healthy habit formation, the more likely they are to adopt it.

Key obstacles to integrating ChatGPT in South African healthcare for children and adolescents with diabetes were also identified. Outdated infrastructure, reliance on legacy systems, and limited internet access, particularly in rural areas, were major barriers. These findings align with Yi et al.^36^ who highlighted inadequate technological infrastructure as a key challenge in AI adoption. Resistance from healthcare professionals was another concern, with participants emphasising that a lack of AI training could lead to scepticism and reluctance to adopt the technology. Moreover, financial constraints were identified as a significant barrier, with participants noting the high cost of integration and limited government support. While previous studies have addressed technological obstacles, this study highlights the critical role of financial constraints and government intervention factors that remain underexplored in existing literature. One of the most important aspects to consider is the alignment of AI-generated recommendations with local clinical protocols. Public healthcare policies in South Africa govern chronic disease management by regulating prescribing practices and clinical decisions based on the Essential Medicines List and code list restrictions. In resource-constrained public sector settings, AI tools such as ChatGPT may generate recommendations that are based on international guidelines or higher-income country standards. In light of this potential mismatch, clinicians must evaluate AI-generated information and ensure that it is aligned with South African policies and regulations, resource availability and situations before applying it in healthcare institutions.

### Limitations

This study has several limitations. As a qualitative study with a small, purposively selected sample, the results are not intended to be statistically generalisable, but rather to provide in-depth, context-specific insights that may be transferable to other contexts. The qualitative approach enabled the researchers to gain a holistic understanding of perceptions, concerns and challenges that may not have been possible to capture through quantitative methods. Future researchers may build on these findings by applying quantitative or mixed methods studies to investigate the prevalence of these perspectives in a broader professional context. Another limitation is that the study did not include insights from AI and IT experts. This omission means that critical technical perspectives on algorithm development, data management and AI integration in healthcare infrastructure were not explored, potentially limiting the depth of analysis regarding the technical implementation of ChatGPT for diabetes management. Future studies should incorporate insights from these experts to gain a deeper understanding of the technical aspects. Furthermore, future studies should consider examining the perspectives of parents, children and adolescents living with diabetes to understand their willingness to use AI tools as part of their self-management routines. Research in this area could focus on the engagement of young patients with the platform, the types of support they expect, and any concerns they may have.

### Recommendations

The study makes the following recommendations:

- The findings revealed that ChatGPT could be a valuable supplementary tool for offering personalised information to children and adolescents with diabetes. Consequently, the study recommends that the South African government, along with policymakers and legislators, establish regulatory and policy frameworks to promote the integration of ChatGPT into clinical practice to support children and adolescents living with diabetes.
- The study recommends that the South African government modernise outdated health systems, update hardware and software, and enhance technological infrastructure in hospitals and clinics to ensure compatibility with ChatGPT for diabetes management.
- The study recommends the need for healthcare professionals and other stakeholders to guide the use of ChatGPT in diabetes management to address concerns about accuracy, ethical use and potential overreliance. This involves teaching patients and caregivers how to evaluate AI-generated information, encouraging them to verify responses with qualified healthcare providers, and integrating ChatGPT instead of replacing clinical judgement with it. Furthermore, health professionals should also advocate for age-appropriate and AI systems that are regulated and clinically validated.
- It is recommended to offer ongoing training programmes for healthcare professionals and parents to understand how AI tools like ChatGPT can be used in clinical settings to support parents of children and adolescents with diabetes.
- Workshops and seminars should be organised to raise public awareness about the potential of ChatGPT, particularly in supporting parents of children and adolescents with diabetes. Furthermore, there is a need to educate and raise awareness among patients, caregivers and healthcare professionals about cybersecurity, data privacy and responsible sharing of personal health information when using ChatGPT.
- The findings suggest that policymakers and other healthcare stakeholders need to work together to develop strategies that effectively address potential challenges hindering the integration of ChatGPT into the South African healthcare.

## Conclusion

Findings revealed the potential of ChatGPT to provide personalised, immediate support but also revealed concerns regarding language barriers, information reliability, data security, privacy, transparency and the risk of overreliance on technology. Key obstacles include outdated infrastructure, legacy health systems, resistance to change among healthcare professionals and insufficient financial resources, which could hinder its successful implementation. This study contributes to the development of policy on AI integration by identifying ethical, infrastructural and regulatory considerations that must be addressed before tools like ChatGPT can be effectively deployed.

Despite the potential of ChatGPT to improve social and educational support, clinical decision-making and medical advice should remain the responsibility of qualified healthcare professionals and practitioners. The study underscores the need for collaboration among healthcare professionals, AI developers, and policymakers to optimise the capabilities of ChatGPT in diabetes management. As ChatGPT advances, its integration into diabetes management may contribute to a more tailored, effective and accessible approach to managing this chronic illness.
